# Supplementary material for: End Sequence Analysis Toolkit (ESAT) expands the extractable information from single-cell RNA-seq data
Source: Genome Res. 2016 Oct;26(10):1397–410. doi: 10.1101/gr.207902.116 (PMC5052061; doi:10.1101/gr.207902.116)
Supplement: Supplemental Material [file supp_gr.207902.116_Supp_Materials.tar.gz › ESAT-esat_v0.1/doc/html/classbroad_1_1pda_1_1annotation_1_1_b_e_d_file_parser.html]

ESAT: broad.pda.annotation.BEDFileParser Class Reference


ESAT


- Main Page
- Related Pages
- Packages
- Classes
- Files

- Class List
- Class Index
- Class Hierarchy
- Class Members

All Classes Namespaces Files Functions Variables Enumerator Pages

- broad
- pda
- annotation
- BEDFileParser

Public Member Functions |
Static Public Member Functions |
Protected Member Functions |
List of all members

broad.pda.annotation.BEDFileParser Class Reference

Inheritance diagram for broad.pda.annotation.BEDFileParser:

[legend]

Collaboration diagram for broad.pda.annotation.BEDFileParser:

[legend]

|  |  |
| --- | --- |
| Public Member Functions | |
|  | BEDFileParser () |
|  | |
|  | BEDFileParser (String fileName) throws IOException |
|  | |
|  | BEDFileParser (String fileName, int ThreePrimeTrimSize) throws IOException |
|  | |
|  | BEDFileParser (String fileName, String chr) throws IOException |
|  | |
|  | BEDFileParser (Collection< Gene > genes) |
|  | |
|  | BEDFileParser (Map< String, Collection< Gene >> transcripts) |
|  | |
| GeneWithIsoforms | get (String geneName) |
|  | |
| List< Gene > | GetGenes () |
|  | |
| List< GeneWithIsoforms > | GetGenesWithIsoforms () |
|  | |
| IntervalTree< GeneWithIsoforms > | getChrTree (String chr) |
|  | |
| Map< String, IntervalTree  < GeneWithIsoforms > > | getIntervalTreeWithIsoforoms () |
|  | |
| Iterator< String > | getChromosomeIterator () |
|  | |
| IntervalTree< GeneWithIsoforms > | getOverlappers (Gene gene) |
|  | |
| IntervalTree< GeneWithIsoforms > | getOverlappers (LightweightGenomicAnnotation region) |
|  | |
| Iterator< GeneWithIsoforms > | getGenomicRegionOverlappers (LightweightGenomicAnnotation region) |
|  | |
| BEDFileParser | getMergedCopy () |
|  | |
| Map< String, IntervalTree  < GeneWithIsoforms > > | getMergedAnnotationMap (double minPctOverlapToMerge) |
|  | |
| Map< String, IntervalTree  < GeneWithIsoforms > > | getMergedAnnotationMap (boolean mergall) |
|  | |
| Map< String, IntervalTree  < GeneWithIsoforms > > | getAnnotationSetMap () |
|  | |
| int | getNumberOfIsoforms (String chr) |
|  | |
| int | getNumberOfIsoforms () |
|  | |
| Gene | getExactIsoform (GeneWithIsoforms loci) |
|  | |
| HashMap< String, Gene > | getNameGeneMap () |
|  | |
| BEDReader | get3PRegion (int toIn, int toOut) |
|  | |
| BEDReader | getPromoters () |
|  | |
| BEDReader | getPromoters (int toIn, int toOut) |
|  | |
| void | loadGTF (File file, String chr) throws IOException |
|  | |
| void | addRefSeq (Gene gene) |
|  | |
| void | addRefSeq (GeneWithIsoforms gene) |
|  | |
| void | addRefSeqSet (Collection<?extends Gene > collection) |
|  | |
| void | writeFullBed (String save) throws IOException |
|  | |
| void | writeFullBed (String save, boolean useExtraFields) throws IOException |
|  | |
| void | writeFullBed (BufferedWriter bw) throws IOException |
|  | |
| void | writeFullBed (BufferedWriter bw, boolean useExtraFields) throws IOException |
|  | |
| void | makeGenes () |
|  | |
| void | makeGenes (double minPctOverlapToMerge) |
|  | |
| boolean | isEmpty () |
|  | |
| boolean | contains (String geneName) |
|  | |
| boolean | containChr (String chr) |
|  | |
| void | clear () |
|  | |
| void | merge () |
|  | |
| void | collapseSingleExonTranscripts () |
|  | |
| void | IncrementScoreIfOverlap (BEDFileParser bed, int setNumber, Boolean setToZero) |
|  | |
| void | updateScrToBedScore (double score) |
|  | |
| void | filterByOverlap (Collection< Gene > genes) |
|  | |
| void | intersect (BEDFileParser bed) |
|  | |
| void | bed2gtf (BufferedWriter bw, String src) throws Exception |
|  | |
| void | bed2CuffGtf (BufferedWriter bw, String src, boolean useExtraFileds) throws Exception |
|  | |
| void | bed2gtf\_bundleOverlappers (BufferedWriter bw, String src, BEDFileParser mergedData) throws IOException |
|  | |
| void | dedup () |
|  | |
| void | minus (Collection< Gene > genes, boolean recursive) |
|  | |
| GeneWithIsoforms | remove (GeneWithIsoforms record) |
|  | |
| Map< String, Collection< Gene > > | toMap () |
|  | |
| Map< String, Collection< Gene > > | toConstituentIsoformMap () |
|  | |
| Map< String, Collection< Gene > > | toConstituentIsoformMap (boolean removeGenesWithoutConstituentIsoforms) |
|  | |
| Map< String, Collection< Gene > > | toConstituentIntroformMap () |
|  | |
| BEDFileParser | copy () |
|  | |
| BEDFileParser | overlap\_GenomicLevel (BEDFileParser other, String task, String considerOrientaion) |
|  | |
| BEDFileParser | overlapByGenomicRegion (BEDFileParser other, String considerOrientaion) |
|  | |
| BEDFileParser | mergeByGtfGeneId () |
|  | |
| BEDFileParser | equalizeTranscriptEnds (BEDFileParser other) |
|  | |
| BEDFileParser | differenceByGenomicRegion (BEDFileParser other, String considerOrientaion) |
|  | |
| BEDFileParser | removeSmallTranscripts (int minExnNum, int minTranscriptLength) |
|  | |
| BEDFileParser | removeSingeleExon () |
|  | |
| GeneWithIsoforms | getClosestUpstream (Gene annot) |
|  | |
| GeneWithIsoforms | getClosestDownstream (Gene annot) |
|  | |
| GeneWithIsoforms | getClosestUpstream (LightweightGenomicAnnotation annot) |
|  | |
| GeneWithIsoforms | getClosestDownstream (LightweightGenomicAnnotation annot) |
|  | |

|  |  |
| --- | --- |
| Static Public Member Functions | |
| static Gene | getGeneByName (String geneName, String bedFile) throws IOException |
|  | |
| static double | getNumberOfIsoforms (IntervalTree< GeneWithIsoforms > overlapTree) |
|  | |
| static Set< Gene > | loadData (File file) throws IOException |
|  | |
| static Set< Gene > | loadData (File file, Alignments region) throws IOException |
|  | |
| static Map< String, Gene > | loadDataByName (File file) throws IOException |
|  | |
| static Map< String, Collection  < Gene > > | loadDataByChr (String file) throws IOException |
|  | |
| static Map< String, Collection  < Gene > > | loadDataByChr (File file) throws IOException |
|  | |
| static Map< String,   IntervalTree< Gene > > | loadDataByChrToTree (File file) throws IOException |
|  | |
| static Map< String, Integer > | loadChrSizes (String file) |
|  | |
| static List< String > | loadList (String file) throws IOException |
|  | |
| static List< String > | loadList (String file, boolean skipFirstLine) throws IOException |
|  | |
| static Map< Alignments, String > | loadDataLine (File file) throws IOException |
|  | |
| static Set< Annotation > | loadAlignmentData (File file) throws IOException |
|  | |
| static Set< Annotation > | loadAlignmentData (BufferedReader reader) throws IOException |
|  | |
| static Set< Annotation > | loadAlignmentData (File file, boolean ucscDownload) throws IOException |
|  | |
| static Map< String, Collection  < Annotation > > | loadAlignmentDataByChr (File file) throws IOException |
|  | |
| static Map< String,   IntervalTree< Alignments > > | loadAlignmentDataToTree (File file) throws IOException |
|  | |
| static Map< String, List  < Alignments > > | loadAlignmentDataByChrArrayList (File file) throws IOException |
|  | |
| static Map< String, Set  < Alignments > > | loadAlignmentDataByChr3Prime (File file, int extensionFactor) throws IOException |
|  | |
| static Map< String, Set  < Alignments > > | loadAlignmentDataByChr5Prime (File file, int extensionFactor) throws IOException |
|  | |
| static Map< Alignments, String > | loadDataLine (BufferedReader reader) throws IOException |
|  | |
| static void | writeBED (String save, Collection< Annotation > regions) throws IOException |
|  | |
| static void | writeBED (String save, Map< Annotation, double[]> scores) throws IOException |
|  | |
| static void | writeSortedBED (String save, Map< Annotation, double[]> scores) throws IOException |
|  | |
| static void | writeSortedBED (String save, Collection< Annotation > alignments) throws IOException |
|  | |
| static void | writeSortedBEDWithScores (String save, Map< Annotation, double[]> scores) throws IOException |
|  | |
| static void | writeBEDWithScores (String save, Map< Annotation, double[]> scores) throws IOException |
|  | |
| static void | writeBED (String save, Collection< Annotation > alignments, boolean sort) throws IOException |
|  | |
| static void | writeBED (String save, Map< Annotation, double[]> scores, boolean withScores, boolean sort) throws IOException |
|  | |
| static void | writeFullBED (String save, Collection< Gene > genes) throws IOException |
|  | |
| static void | writeFullBED (String save, Map< Gene,?extends Object > genes) throws IOException |
|  | |
| static Map< String,   IntervalTree< Gene > > | makeIntervalTreeFor3Prime (File geneFile, int size) throws IOException |
|  | |
| static boolean | isOverlapCompatible (Gene first, Gene second, double minPctOverlap) |
|  | |
| static void | main (String[] args) throws IOException |
|  | |
| static Map< String, BEDFileParser > | uploadSetOfFiles (String bedLst) throws IOException |
|  | |
| static BEDFileParser | expanedUtrs (BEDFileParser bed, Integer utr1, Integer utr2) |
|  | |
| static BEDFileParser | readSiphyOutToBed (String siphyFile) throws NumberFormatException, IOException |
|  | |
| static Gene | getGene (String geneFile, String geneName) throws IOException |
|  | |

|  |  |
| --- | --- |
| Protected Member Functions | |
| void | loadIsoformDataByChrToTree (File file) throws IOException |
|  | |
| void | loadIsoformDataByChrToTree (File file, String chr) throws IOException |
|  | |

## Constructor & Destructor Documentation

|  |  |  |  |  |
| --- | --- | --- | --- | --- |
| broad.pda.annotation.BEDFileParser.BEDFileParser | ( |  | ) |  |

Here is the caller graph for this function:

|  |  |  |  |  |  |
| --- | --- | --- | --- | --- | --- |
| broad.pda.annotation.BEDFileParser.BEDFileParser | ( | String | *fileName* | ) | throws IOException |

Here is the call graph for this function:

|  |  |  |  |
| --- | --- | --- | --- |
| broad.pda.annotation.BEDFileParser.BEDFileParser | ( | String | *fileName*, |
|  |  | int | *ThreePrimeTrimSize* |
|  | ) |  | throws IOException |

|  |  |  |  |
| --- | --- | --- | --- |
| broad.pda.annotation.BEDFileParser.BEDFileParser | ( | String | *fileName*, |
|  |  | String | *chr* |
|  | ) |  | throws IOException |

Here is the call graph for this function:

|  |  |  |  |  |  |
| --- | --- | --- | --- | --- | --- |
| broad.pda.annotation.BEDFileParser.BEDFileParser | ( | Collection< Gene > | *genes* | ) |  |

Here is the call graph for this function:

|  |  |  |  |  |  |
| --- | --- | --- | --- | --- | --- |
| broad.pda.annotation.BEDFileParser.BEDFileParser | ( | Map< String, Collection< Gene >> | *transcripts* | ) |  |

Here is the call graph for this function:

## Member Function Documentation

|  |  |  |  |  |  |
| --- | --- | --- | --- | --- | --- |
| void broad.pda.annotation.BEDFileParser.addRefSeq | ( | Gene | *gene* | ) |  |

Here is the call graph for this function:

Here is the caller graph for this function:

|  |  |  |  |  |  |
| --- | --- | --- | --- | --- | --- |
| void broad.pda.annotation.BEDFileParser.addRefSeq | ( | GeneWithIsoforms | *gene* | ) |  |

Here is the call graph for this function:

|  |  |  |  |  |  |
| --- | --- | --- | --- | --- | --- |
| void broad.pda.annotation.BEDFileParser.addRefSeqSet | ( | Collection<?extends Gene > | *collection* | ) |  |

Here is the caller graph for this function:

|  |  |  |  |
| --- | --- | --- | --- |
| void broad.pda.annotation.BEDFileParser.bed2CuffGtf | ( | BufferedWriter | *bw*, |
|  |  | String | *src*, |
|  |  | boolean | *useExtraFileds* |
|  | ) |  | throws Exception |

Here is the call graph for this function:

Here is the caller graph for this function:

|  |  |  |  |
| --- | --- | --- | --- |
| void broad.pda.annotation.BEDFileParser.bed2gtf | ( | BufferedWriter | *bw*, |
|  |  | String | *src* |
|  | ) |  | throws Exception |

Here is the caller graph for this function:

|  |  |  |  |
| --- | --- | --- | --- |
| void broad.pda.annotation.BEDFileParser.bed2gtf\_bundleOverlappers | ( | BufferedWriter | *bw*, |
|  |  | String | *src*, |
|  |  | BEDFileParser | *mergedData* |
|  | ) |  | throws IOException |

Here is the call graph for this function:

|  |  |  |  |  |
| --- | --- | --- | --- | --- |
| void broad.pda.annotation.BEDFileParser.clear | ( |  | ) |  |

|  |  |  |  |  |
| --- | --- | --- | --- | --- |
| void broad.pda.annotation.BEDFileParser.collapseSingleExonTranscripts | ( |  | ) |  |

Merges all single exon transcripts

Here is the call graph for this function:

|  |  |  |  |  |  |
| --- | --- | --- | --- | --- | --- |
| boolean broad.pda.annotation.BEDFileParser.containChr | ( | String | *chr* | ) |  |

Here is the caller graph for this function:

|  |  |  |  |  |  |
| --- | --- | --- | --- | --- | --- |
| boolean broad.pda.annotation.BEDFileParser.contains | ( | String | *geneName* | ) |  |

|  |  |  |  |  |
| --- | --- | --- | --- | --- |
| BEDFileParser broad.pda.annotation.BEDFileParser.copy | ( |  | ) |  |

Here is the call graph for this function:

|  |  |  |  |  |
| --- | --- | --- | --- | --- |
| void broad.pda.annotation.BEDFileParser.dedup | ( |  | ) |  |

|  |  |  |  |
| --- | --- | --- | --- |
| BEDFileParser broad.pda.annotation.BEDFileParser.differenceByGenomicRegion | ( | BEDFileParser | *other*, |
|  |  | String | *considerOrientaion* |
|  | ) |  |  |

|  |  |  |  |  |  |
| --- | --- | --- | --- | --- | --- |
| BEDFileParser broad.pda.annotation.BEDFileParser.equalizeTranscriptEnds | ( | BEDFileParser | *other* | ) |  |

|  |  |  |  |
| --- | --- | --- | --- |
| static BEDFileParser broad.pda.annotation.BEDFileParser.expanedUtrs | ( | BEDFileParser | *bed*, |
|  |  | Integer | *utr1*, |
|  |  | Integer | *utr2* |
|  | ) |  |  |

static

Here is the call graph for this function:

|  |  |  |  |  |  |
| --- | --- | --- | --- | --- | --- |
| void broad.pda.annotation.BEDFileParser.filterByOverlap | ( | Collection< Gene > | *genes* | ) |  |

|  |  |  |  |  |  |
| --- | --- | --- | --- | --- | --- |
| GeneWithIsoforms broad.pda.annotation.BEDFileParser.get | ( | String | *geneName* | ) |  |

Here is the caller graph for this function:

|  |  |  |  |
| --- | --- | --- | --- |
| BEDReader broad.pda.annotation.BEDFileParser.get3PRegion | ( | int | *toIn*, |
|  |  | int | *toOut* |
|  | ) |  |  |

Here is the call graph for this function:

|  |  |  |  |  |
| --- | --- | --- | --- | --- |
| Map<String, IntervalTree<GeneWithIsoforms> > broad.pda.annotation.BEDFileParser.getAnnotationSetMap | ( |  | ) |  |

|  |  |  |  |  |
| --- | --- | --- | --- | --- |
| Iterator<String> broad.pda.annotation.BEDFileParser.getChromosomeIterator | ( |  | ) |  |

Here is the caller graph for this function:

|  |  |  |  |  |  |
| --- | --- | --- | --- | --- | --- |
| IntervalTree<GeneWithIsoforms> broad.pda.annotation.BEDFileParser.getChrTree | ( | String | *chr* | ) |  |

Here is the caller graph for this function:

|  |  |  |  |  |  |
| --- | --- | --- | --- | --- | --- |
| GeneWithIsoforms broad.pda.annotation.BEDFileParser.getClosestDownstream | ( | Gene | *annot* | ) |  |

Here is the call graph for this function:

|  |  |  |  |  |  |
| --- | --- | --- | --- | --- | --- |
| GeneWithIsoforms broad.pda.annotation.BEDFileParser.getClosestDownstream | ( | LightweightGenomicAnnotation | *annot* | ) |  |

Here is the call graph for this function:

|  |  |  |  |  |  |
| --- | --- | --- | --- | --- | --- |
| GeneWithIsoforms broad.pda.annotation.BEDFileParser.getClosestUpstream | ( | Gene | *annot* | ) |  |

Here is the call graph for this function:

|  |  |  |  |  |  |
| --- | --- | --- | --- | --- | --- |
| GeneWithIsoforms broad.pda.annotation.BEDFileParser.getClosestUpstream | ( | LightweightGenomicAnnotation | *annot* | ) |  |

Here is the call graph for this function:

|  |  |  |  |  |  |
| --- | --- | --- | --- | --- | --- |
| Gene broad.pda.annotation.BEDFileParser.getExactIsoform | ( | GeneWithIsoforms | *loci* | ) |  |

|  |  |  |  |
| --- | --- | --- | --- |
| static Gene broad.pda.annotation.BEDFileParser.getGene | ( | String | *geneFile*, |
|  |  | String | *geneName* |
|  | ) |  | throws IOException |

static

|  |  |  |  |
| --- | --- | --- | --- |
| static Gene broad.pda.annotation.BEDFileParser.getGeneByName | ( | String | *geneName*, |
|  |  | String | *bedFile* |
|  | ) |  | throws IOException |

static

Get gene from bed file by gene name

Parameters
:   |  |  |
    | --- | --- |
    | geneName | Gene name |
    | bedFile | Bed file |

Returns
:   Gene model described by the first line in the bed file with name field equal to gene name

Exceptions
:   |  |  |
    | --- | --- |
    | IOException |  |

Here is the call graph for this function:

|  |  |  |  |  |
| --- | --- | --- | --- | --- |
| List<Gene> broad.pda.annotation.BEDFileParser.GetGenes | ( |  | ) |  |

Here is the caller graph for this function:

|  |  |  |  |  |
| --- | --- | --- | --- | --- |
| List<GeneWithIsoforms> broad.pda.annotation.BEDFileParser.GetGenesWithIsoforms | ( |  | ) |  |

|  |  |  |  |  |  |
| --- | --- | --- | --- | --- | --- |
| Iterator<GeneWithIsoforms> broad.pda.annotation.BEDFileParser.getGenomicRegionOverlappers | ( | LightweightGenomicAnnotation | *region* | ) |  |

Here is the call graph for this function:

|  |  |  |  |  |
| --- | --- | --- | --- | --- |
| Map<String, IntervalTree<GeneWithIsoforms> > broad.pda.annotation.BEDFileParser.getIntervalTreeWithIsoforoms | ( |  | ) |  |

|  |  |  |  |  |  |
| --- | --- | --- | --- | --- | --- |
| Map<String, IntervalTree<GeneWithIsoforms> > broad.pda.annotation.BEDFileParser.getMergedAnnotationMap | ( | double | *minPctOverlapToMerge* | ) |  |

Here is the call graph for this function:

Here is the caller graph for this function:

|  |  |  |  |  |  |
| --- | --- | --- | --- | --- | --- |
| Map<String, IntervalTree<GeneWithIsoforms> > broad.pda.annotation.BEDFileParser.getMergedAnnotationMap | ( | boolean | *mergall* | ) |  |

Here is the call graph for this function:

|  |  |  |  |  |
| --- | --- | --- | --- | --- |
| BEDFileParser broad.pda.annotation.BEDFileParser.getMergedCopy | ( |  | ) |  |

Here is the call graph for this function:

|  |  |  |  |  |
| --- | --- | --- | --- | --- |
| HashMap<String, Gene> broad.pda.annotation.BEDFileParser.getNameGeneMap | ( |  | ) |  |

Here is the call graph for this function:

|  |  |  |  |  |  |
| --- | --- | --- | --- | --- | --- |
| int broad.pda.annotation.BEDFileParser.getNumberOfIsoforms | ( | String | *chr* | ) |  |

|  |  |  |  |  |
| --- | --- | --- | --- | --- |
| int broad.pda.annotation.BEDFileParser.getNumberOfIsoforms | ( |  | ) |  |

Returns
:   Number of transcripts in the reader

Here is the call graph for this function:

|  |  |  |  |  |  |
| --- | --- | --- | --- | --- | --- |
| static double broad.pda.annotation.BEDFileParser.getNumberOfIsoforms | ( | IntervalTree< GeneWithIsoforms > | *overlapTree* | ) |  |

static

|  |  |  |  |  |  |
| --- | --- | --- | --- | --- | --- |
| IntervalTree<GeneWithIsoforms> broad.pda.annotation.BEDFileParser.getOverlappers | ( | Gene | *gene* | ) |  |

Here is the call graph for this function:

Here is the caller graph for this function:

|  |  |  |  |  |  |
| --- | --- | --- | --- | --- | --- |
| IntervalTree<GeneWithIsoforms> broad.pda.annotation.BEDFileParser.getOverlappers | ( | LightweightGenomicAnnotation | *region* | ) |  |

Here is the call graph for this function:

|  |  |  |  |  |
| --- | --- | --- | --- | --- |
| BEDReader broad.pda.annotation.BEDFileParser.getPromoters | ( |  | ) |  |

|  |  |  |  |
| --- | --- | --- | --- |
| BEDReader broad.pda.annotation.BEDFileParser.getPromoters | ( | int | *toIn*, |
|  |  | int | *toOut* |
|  | ) |  |  |

Here is the call graph for this function:

|  |  |  |  |
| --- | --- | --- | --- |
| void broad.pda.annotation.BEDFileParser.IncrementScoreIfOverlap | ( | BEDFileParser | *bed*, |
|  |  | int | *setNumber*, |
|  |  | Boolean | *setToZero* |
|  | ) |  |  |

Here is the call graph for this function:

|  |  |  |  |  |  |
| --- | --- | --- | --- | --- | --- |
| void broad.pda.annotation.BEDFileParser.intersect | ( | BEDFileParser | *bed* | ) |  |

|  |  |  |  |  |
| --- | --- | --- | --- | --- |
| boolean broad.pda.annotation.BEDFileParser.isEmpty | ( |  | ) |  |

|  |  |  |  |
| --- | --- | --- | --- |
| static boolean broad.pda.annotation.BEDFileParser.isOverlapCompatible | ( | Gene | *first*, |
|  |  | Gene | *second*, |
|  |  | double | *minPctOverlap* |
|  | ) |  |  |

static

Here is the call graph for this function:

Here is the caller graph for this function:

|  |  |  |  |  |  |
| --- | --- | --- | --- | --- | --- |
| static Set<Annotation> broad.pda.annotation.BEDFileParser.loadAlignmentData | ( | File | *file* | ) | throws IOException |

static

Here is the caller graph for this function:

|  |  |  |  |  |  |
| --- | --- | --- | --- | --- | --- |
| static Set<Annotation> broad.pda.annotation.BEDFileParser.loadAlignmentData | ( | BufferedReader | *reader* | ) | throws IOException |

static

|  |  |  |  |
| --- | --- | --- | --- |
| static Set<Annotation> broad.pda.annotation.BEDFileParser.loadAlignmentData | ( | File | *file*, |
|  |  | boolean | *ucscDownload* |
|  | ) |  | throws IOException |

static

Here is the call graph for this function:

|  |  |  |  |  |  |
| --- | --- | --- | --- | --- | --- |
| static Map<String, Collection<Annotation> > broad.pda.annotation.BEDFileParser.loadAlignmentDataByChr | ( | File | *file* | ) | throws IOException |

static

|  |  |  |  |
| --- | --- | --- | --- |
| static Map<String, Set<Alignments> > broad.pda.annotation.BEDFileParser.loadAlignmentDataByChr3Prime | ( | File | *file*, |
|  |  | int | *extensionFactor* |
|  | ) |  | throws IOException |

static

|  |  |  |  |
| --- | --- | --- | --- |
| static Map<String, Set<Alignments> > broad.pda.annotation.BEDFileParser.loadAlignmentDataByChr5Prime | ( | File | *file*, |
|  |  | int | *extensionFactor* |
|  | ) |  | throws IOException |

static

|  |  |  |  |  |  |
| --- | --- | --- | --- | --- | --- |
| static Map<String, List<Alignments> > broad.pda.annotation.BEDFileParser.loadAlignmentDataByChrArrayList | ( | File | *file* | ) | throws IOException |

static

|  |  |  |  |  |  |
| --- | --- | --- | --- | --- | --- |
| static Map<String, IntervalTree<Alignments> > broad.pda.annotation.BEDFileParser.loadAlignmentDataToTree | ( | File | *file* | ) | throws IOException |

static

|  |  |  |  |  |  |
| --- | --- | --- | --- | --- | --- |
| static Map<String, Integer> broad.pda.annotation.BEDFileParser.loadChrSizes | ( | String | *file* | ) |  |

static

Here is the caller graph for this function:

|  |  |  |  |  |  |
| --- | --- | --- | --- | --- | --- |
| static Set<Gene> broad.pda.annotation.BEDFileParser.loadData | ( | File | *file* | ) | throws IOException |

static

Here is the caller graph for this function:

|  |  |  |  |
| --- | --- | --- | --- |
| static Set<Gene> broad.pda.annotation.BEDFileParser.loadData | ( | File | *file*, |
|  |  | Alignments | *region* |
|  | ) |  | throws IOException |

static

Here is the call graph for this function:

|  |  |  |  |  |  |
| --- | --- | --- | --- | --- | --- |
| static Map<String, Collection<Gene> > broad.pda.annotation.BEDFileParser.loadDataByChr | ( | String | *file* | ) | throws IOException |

static

Read genes from the bed file and get genes by chromosome

Parameters
:   |  |  |
    | --- | --- |
    | file | Name of bed file |

Returns
:   Map of chromosome name to set of genes on chromosome

Exceptions
:   |  |  |
    | --- | --- |
    | IOException |  |

Here is the caller graph for this function:

|  |  |  |  |  |  |
| --- | --- | --- | --- | --- | --- |
| static Map<String, Collection<Gene> > broad.pda.annotation.BEDFileParser.loadDataByChr | ( | File | *file* | ) | throws IOException |

static

Here is the call graph for this function:

|  |  |  |  |  |  |
| --- | --- | --- | --- | --- | --- |
| static Map<String, IntervalTree<Gene> > broad.pda.annotation.BEDFileParser.loadDataByChrToTree | ( | File | *file* | ) | throws IOException |

static

Here is the call graph for this function:

|  |  |  |  |  |  |
| --- | --- | --- | --- | --- | --- |
| static Map<String, Gene> broad.pda.annotation.BEDFileParser.loadDataByName | ( | File | *file* | ) | throws IOException |

static

|  |  |  |  |  |  |
| --- | --- | --- | --- | --- | --- |
| static Map<Alignments, String> broad.pda.annotation.BEDFileParser.loadDataLine | ( | File | *file* | ) | throws IOException |

static

|  |  |  |  |  |  |
| --- | --- | --- | --- | --- | --- |
| static Map<Alignments, String> broad.pda.annotation.BEDFileParser.loadDataLine | ( | BufferedReader | *reader* | ) | throws IOException |

static

|  |  |  |  |
| --- | --- | --- | --- |
| void broad.pda.annotation.BEDFileParser.loadGTF | ( | File | *file*, |
|  |  | String | *chr* |
|  | ) |  | throws IOException |

|  |  |  |  |  |  |
| --- | --- | --- | --- | --- | --- |
| void broad.pda.annotation.BEDFileParser.loadIsoformDataByChrToTree | ( | File | *file* | ) | throws IOException |

protected

Here is the caller graph for this function:

|  |  |  |  |
| --- | --- | --- | --- |
| void broad.pda.annotation.BEDFileParser.loadIsoformDataByChrToTree | ( | File | *file*, |
|  |  | String | *chr* |
|  | ) |  | throws IOException |

protected

|  |  |  |  |  |  |
| --- | --- | --- | --- | --- | --- |
| static List<String> broad.pda.annotation.BEDFileParser.loadList | ( | String | *file* | ) | throws IOException |

static

|  |  |  |  |
| --- | --- | --- | --- |
| static List<String> broad.pda.annotation.BEDFileParser.loadList | ( | String | *file*, |
|  |  | boolean | *skipFirstLine* |
|  | ) |  | throws IOException |

static

|  |  |  |  |  |  |
| --- | --- | --- | --- | --- | --- |
| static void broad.pda.annotation.BEDFileParser.main | ( | String[] | *args* | ) | throws IOException |

static

Here is the call graph for this function:

|  |  |  |  |  |
| --- | --- | --- | --- | --- |
| void broad.pda.annotation.BEDFileParser.makeGenes | ( |  | ) |  |

|  |  |  |  |  |  |
| --- | --- | --- | --- | --- | --- |
| void broad.pda.annotation.BEDFileParser.makeGenes | ( | double | *minPctOverlapToMerge* | ) |  |

Here is the call graph for this function:

|  |  |  |  |
| --- | --- | --- | --- |
| static Map<String, IntervalTree<Gene> > broad.pda.annotation.BEDFileParser.makeIntervalTreeFor3Prime | ( | File | *geneFile*, |
|  |  | int | *size* |
|  | ) |  | throws IOException |

static

Here is the call graph for this function:

|  |  |  |  |  |
| --- | --- | --- | --- | --- |
| void broad.pda.annotation.BEDFileParser.merge | ( |  | ) |  |

Here is the call graph for this function:

|  |  |  |  |  |
| --- | --- | --- | --- | --- |
| BEDFileParser broad.pda.annotation.BEDFileParser.mergeByGtfGeneId | ( |  | ) |  |

Here is the call graph for this function:

|  |  |  |  |
| --- | --- | --- | --- |
| void broad.pda.annotation.BEDFileParser.minus | ( | Collection< Gene > | *genes*, |
|  |  | boolean | *recursive* |
|  | ) |  |  |

Removes from this reader all elements that overlap the given list.

Parameters
:   |  |  |
    | --- | --- |
    | recursive | - if true not only transcripts overlapping the given set but also transcripts that overlap the overlappers will be removed |

Here is the call graph for this function:

|  |  |  |  |
| --- | --- | --- | --- |
| BEDFileParser broad.pda.annotation.BEDFileParser.overlap\_GenomicLevel | ( | BEDFileParser | *other*, |
|  |  | String | *task*, |
|  |  | String | *considerOrientaion* |
|  | ) |  |  |

Here is the call graph for this function:

|  |  |  |  |
| --- | --- | --- | --- |
| BEDFileParser broad.pda.annotation.BEDFileParser.overlapByGenomicRegion | ( | BEDFileParser | *other*, |
|  |  | String | *considerOrientaion* |
|  | ) |  |  |

|  |  |  |  |  |  |
| --- | --- | --- | --- | --- | --- |
| static BEDFileParser broad.pda.annotation.BEDFileParser.readSiphyOutToBed | ( | String | *siphyFile* | ) | throws NumberFormatException, IOException |

static

Here is the call graph for this function:

|  |  |  |  |  |  |
| --- | --- | --- | --- | --- | --- |
| GeneWithIsoforms broad.pda.annotation.BEDFileParser.remove | ( | GeneWithIsoforms | *record* | ) |  |

Here is the call graph for this function:

|  |  |  |  |  |
| --- | --- | --- | --- | --- |
| BEDFileParser broad.pda.annotation.BEDFileParser.removeSingeleExon | ( |  | ) |  |

Here is the call graph for this function:

|  |  |  |  |
| --- | --- | --- | --- |
| BEDFileParser broad.pda.annotation.BEDFileParser.removeSmallTranscripts | ( | int | *minExnNum*, |
|  |  | int | *minTranscriptLength* |
|  | ) |  |  |

Here is the call graph for this function:

|  |  |  |  |  |
| --- | --- | --- | --- | --- |
| Map<String, Collection<Gene> > broad.pda.annotation.BEDFileParser.toConstituentIntroformMap | ( |  | ) |  |

Builds an artificial map of isoforms where "constituent" introns are the exons.

Returns

|  |  |  |  |  |
| --- | --- | --- | --- | --- |
| Map<String, Collection<Gene> > broad.pda.annotation.BEDFileParser.toConstituentIsoformMap | ( |  | ) |  |

|  |  |  |  |  |  |
| --- | --- | --- | --- | --- | --- |
| Map<String, Collection<Gene> > broad.pda.annotation.BEDFileParser.toConstituentIsoformMap | ( | boolean | *removeGenesWithoutConstituentIsoforms* | ) |  |

|  |  |  |  |  |
| --- | --- | --- | --- | --- |
| Map<String, Collection<Gene> > broad.pda.annotation.BEDFileParser.toMap | ( |  | ) |  |

|  |  |  |  |  |  |
| --- | --- | --- | --- | --- | --- |
| void broad.pda.annotation.BEDFileParser.updateScrToBedScore | ( | double | *score* | ) |  |

|  |  |  |  |  |  |
| --- | --- | --- | --- | --- | --- |
| static Map<String, BEDFileParser> broad.pda.annotation.BEDFileParser.uploadSetOfFiles | ( | String | *bedLst* | ) | throws IOException |

static

Here is the call graph for this function:

|  |  |  |  |
| --- | --- | --- | --- |
| static void broad.pda.annotation.BEDFileParser.writeBED | ( | String | *save*, |
|  |  | Collection< Annotation > | *regions* |
|  | ) |  | throws IOException |

static

Here is the caller graph for this function:

|  |  |  |  |
| --- | --- | --- | --- |
| static void broad.pda.annotation.BEDFileParser.writeBED | ( | String | *save*, |
|  |  | Map< Annotation, double[]> | *scores* |
|  | ) |  | throws IOException |

static

Here is the call graph for this function:

|  |  |  |  |
| --- | --- | --- | --- |
| static void broad.pda.annotation.BEDFileParser.writeBED | ( | String | *save*, |
|  |  | Collection< Annotation > | *alignments*, |
|  |  | boolean | *sort* |
|  | ) |  | throws IOException |

static

Here is the call graph for this function:

|  |  |  |  |
| --- | --- | --- | --- |
| static void broad.pda.annotation.BEDFileParser.writeBED | ( | String | *save*, |
|  |  | Map< Annotation, double[]> | *scores*, |
|  |  | boolean | *withScores*, |
|  |  | boolean | *sort* |
|  | ) |  | throws IOException |

static

|  |  |  |  |
| --- | --- | --- | --- |
| static void broad.pda.annotation.BEDFileParser.writeBEDWithScores | ( | String | *save*, |
|  |  | Map< Annotation, double[]> | *scores* |
|  | ) |  | throws IOException |

static

Here is the call graph for this function:

|  |  |  |  |  |  |
| --- | --- | --- | --- | --- | --- |
| void broad.pda.annotation.BEDFileParser.writeFullBed | ( | String | *save* | ) | throws IOException |

Here is the caller graph for this function:

|  |  |  |  |
| --- | --- | --- | --- |
| void broad.pda.annotation.BEDFileParser.writeFullBed | ( | String | *save*, |
|  |  | boolean | *useExtraFields* |
|  | ) |  | throws IOException |

Here is the call graph for this function:

|  |  |  |  |  |  |
| --- | --- | --- | --- | --- | --- |
| void broad.pda.annotation.BEDFileParser.writeFullBed | ( | BufferedWriter | *bw* | ) | throws IOException |

Here is the call graph for this function:

|  |  |  |  |
| --- | --- | --- | --- |
| void broad.pda.annotation.BEDFileParser.writeFullBed | ( | BufferedWriter | *bw*, |
|  |  | boolean | *useExtraFields* |
|  | ) |  | throws IOException |

Here is the call graph for this function:

|  |  |  |  |
| --- | --- | --- | --- |
| static void broad.pda.annotation.BEDFileParser.writeFullBED | ( | String | *save*, |
|  |  | Collection< Gene > | *genes* |
|  | ) |  | throws IOException |

static

|  |  |  |  |
| --- | --- | --- | --- |
| static void broad.pda.annotation.BEDFileParser.writeFullBED | ( | String | *save*, |
|  |  | Map< Gene,?extends Object > | *genes* |
|  | ) |  | throws IOException |

static

|  |  |  |  |
| --- | --- | --- | --- |
| static void broad.pda.annotation.BEDFileParser.writeSortedBED | ( | String | *save*, |
|  |  | Map< Annotation, double[]> | *scores* |
|  | ) |  | throws IOException |

static

Here is the call graph for this function:

|  |  |  |  |
| --- | --- | --- | --- |
| static void broad.pda.annotation.BEDFileParser.writeSortedBED | ( | String | *save*, |
|  |  | Collection< Annotation > | *alignments* |
|  | ) |  | throws IOException |

static

Here is the call graph for this function:

|  |  |  |  |
| --- | --- | --- | --- |
| static void broad.pda.annotation.BEDFileParser.writeSortedBEDWithScores | ( | String | *save*, |
|  |  | Map< Annotation, double[]> | *scores* |
|  | ) |  | throws IOException |

static

Here is the call graph for this function:

---

The documentation for this class was generated from the following file:

- java/broad/pda/annotation/BEDFileParser.java


---

Generated on Fri Aug 15 2014 15:10:58 for ESAT by  

 1.8.7
